# Development and validation of nomograms to predict frailty-worsening trajectories among Chinese older adults

**DOI:** 10.3389/fpubh.2025.1588303

**Published:** 2025-07-17

**Authors:** Jiaolan Du, Feng Ye, Min Zhang, Jinping Zeng, Ting Duan, Qin Song, Jun Yang, Yinyin Wu

**Affiliations:** ^1^Department of Epidemiology and Health Statistics, School of Public Health, Hangzhou Normal University, Hangzhou, China; ^2^School of Pharmacy, Hangzhou Normal University, Hangzhou, China; ^3^Department of Occupational and Environmental Health, School of Public Health, Hangzhou Normal University, Hangzhou, China; ^4^Department of Nutrition and Toxicology, School of Public Health, Hangzhou Normal University, Hangzhou, China

**Keywords:** frailty, trajectory, nomogram, prediction, validation

## Abstract

**Background:**

Frailty progression may lead to adverse clinical events. Timely intervention of individual with heterogeneous frailty trajectories are important to prevent or reverse frailty progression.

**Aims:**

This study aimed to develop nomograms to predict heterogeneous frailty progression, and validate their predictive performance.

**Methods:**

4,406 participants (2,268 in the development cohort and 2,138 in the validation cohort) were included in this study. Latent class trajectory model (LCTM) was used to identify the heterogeneous frailty trajectories. Lasso regression analysis was employed to screen predictive factors. The nomogram models were subsequently developed using multivariable logistic regression analysis. Model performance was internally validated with bootstrap resampling and externally validated using independent data. The discrimination and calibration were assessed by C-index and calibration curve, respectively.

**Results:**

Two prediction nomograms were developed and validated to estimate the risk of future frailty progression based on three identified frailty trajectories. Eleven predictors were determined in the medium-stable nomogram. The internal and external validation C-indices were 0.86 and 0.77; the calibration curves demonstrated that the predicted probabilities fit well with the actual observation. Six predictors were determined in the low-rapid nomogram. The internal and external validation C-indices were 0.74 and 0.62, respectively, and calibration curves indicated good calibration.

**Discussion:**

Frailty trajectories provide more predictive value than frailty states. This study developed nomogram models to predict frailty progression, identifying key predictors such as gender, cognitive impairment, lifestyle factors, and early life experiences, with promising validation results.

**Conclusion:**

The nomograms demonstrated favorable performance and may help making public health strategies for more precise frailty management.

## 1 Introduction

Frailty is characterized by the progressive decline of multiple physiological systems, resulting in increased vulnerability to external stressors among older adults (1, 2). Frailty is associated with many adverse health outcomes, including falls, cognitive impairment, fractures, disability, death, and high health expenditure (3–5). As such, frailty is becoming a significant challenge in global public health, especially for China with its large aging population (6).

It is worth noting that frailty is dynamic (7) and each individual probably has unique trajectory of frailty progression (8). In our previous study (9), three frailty trajectories were identified based on a large Chinese population cohort: the low-stable group, the medium-stable group, and the low-rapid group. The latter two trajectories indicated persistent or worsening frailty and were referred to as the “heterogeneous frailty trajectories”. Heterogeneous frailty trajectories also predict other adverse health outcomes and impact individual’s quality of life (10). Early identification and timely intervention of individual with heterogeneous frailty trajectories are important to prevent or reverse frailty progression (11). Thus, there is an urgent need to develop feasible risk prediction models based on frailty trajectories in older adults. The nomogram can visualize statistical predictive models and intuitively generate predictive probabilities of clinical events (12), which facilitates personalized predictions and interventions.

Therefore, this study aimed to develop and validate prediction nomograms for predicting the risk of worsening frailty progression in Chinese older adults, which would be of important public health value.

## 2 Materials and methods

### 2.1 Study participants

#### 2.1.1 Development cohort

Chinese Longitudinal Healthy Longevity Survey (CLHLS) is the first longitudinal survey to investigate the determinants of health and longevity of older adults in China (13). Inclusion criteria for this study were as follows: (1) age 65 years and older; (2) participation in four surveys successively since 2008; (3) completion of baseline frailty measurement and three follow-up frailty measurements. Participants with more than 25% missing data in the frailty assessment were excluded.

#### 2.1.2 Validation cohort

China Health and Retirement Longitudinal Study (CHARLS) (14) is also a nationally representative longitudinal survey designed to collect information on socioeconomic status and personal health status. The CHARLS database covers data from individuals aged 45 years or older and their spouses living in China between 2008 and 2018. Data from the CHARLS was used for external validation in this study. Curation of CHARLS data followed the same process that was done for CLHLS.

The two databases were approved by the Research Ethics Committee of Peking University (IRB00001052-13074 and IRB00001052-11015) and all participants provided written informed consent. Finally, 4,406 participants (2,268 in the development cohort and 2,138 in the validation cohort) were included (Supplementary Figure 1).

### 2.2 Frailty status

Frailty index (FI) was used to measure the dimensions of frailty. Based on our previous study (9), 38 health deficits in the CLHLS and 34 health deficits in the CHARLS were included to construct the FI. FI score was calculated in each wave of both cohorts, respectively. Frailty measurements from baseline to the last follow-up were subjected to trajectory analysis. Detailed deficits of the FI were shown in Supplementary Table 1.

### 2.3 Covariates

Life course theory posits that early life exposure may affect late-life health (15). Thus, we explored possible factors from a full life cycle perspective, including age, gender, education, economic status, health status and behaviors, lifestyle, family social support, and childhood experiences. For the consistency between CLHLS and CHARLS, education level was defined as illiterate (no education) or literate (having years of education). Some common leisure activities were included based on typical practices in Chinese culture, such as gardening, watching TV, and playing mah-jongg. The frequency of these activities were categorized as low-frequency and high-frequency. Information on childhood experiences including losing parents (yes/no), childhood starvation (yes/no), and medical service in childhood (inadequate/adequate) were collected. The level of loneliness was measured by a single-item question on how often the respondent felt lonely and was divided into two categories based on reported frequency (16). A more detailed description of the variables was shown in Supplementary Table 2.

### 2.4 Statistical analysis

For descriptive statistics, multiple imputation was used to interpolate for missing data on continuous variables if the percentage of missing data was <10%. Continuous variables were expressed as mean (M) ± standard deviation (SD) and compared using two-tailed *t*-tests or Kruskal-Wallis rank sum tests. Categorical variables were presented as numbers and proportions, and compared using a chi-square test or Fisher exact test.

Latent class trajectory model (LCTM) was used to identify potential frailty trajectories. The optimal number of trajectories was determined based on several criteria: the lowest Bayesian Information Criterion (BIC) value, an average posterior probability greater than 70%, and each class comprising at least 5% of the total sample. We employed the least absolute shrinkage and selection operator (Lasso) regression analysis and 10-fold cross-validation techniques to identify potential predictive factors (17, 18). Lasso can eliminate coefficients of less important variables and evaluate the significant correlations between independent variables and the outcome. Subsequently, a nomogram prediction model was developed using multivariate logistic regression analysis. A score was assigned to each predictor in the nomogram so that total points could be ascertained to estimate the probability of being in worsening frailty trajectories (19). Meanwhile, we constructed a web-based dynamic online nomogram to facilitate practical application.

The performances of the nomogram were validated in the internal and external validation cohorts concerning discrimination and calibration (20). Internal validation was performed in the development cohort using 1,000 bootstrap resamples, while external validation was conducted using an independent CHARLS cohort. The discrimination performances of the nomogram were assessed by the concordance index (C-index) or the area under curve (AUC) of the receiver operating characteristic (ROC) curve. The AUC value ranges from 0.5 to 1, with value 1 being the best discriminatory ability (21). Calibration curve analysis was conducted to assess the calibration effect by evaluating the consistency of predicted probabilities and observed frequencies.

Statistical analyses and visualizations were performed with R version 4.3.0 (22). All tests were two-sided, and a *p*-value < 0.05 was considered statistically significant.

## 3 Results

### 3.1 Baseline characteristics of the participants

There were statistically significant differences between the development and validation cohorts in the demographical and behavioral characteristics. Compared to the validation cohort, the participants in the development cohort were relatively older (*p* < 0.001). Nevertheless, older adults in both cohorts exhibited a similarly low risk of frailty at baseline. More details were described in Table 1.

**Table 1:** General characteristics of study populations.

Variable	Development cohort (*n* = 2,268)	Validation cohort (*n* = 2,138)	*p* value
Age (mean ± SD)	75.46 ± 8.03	70.41 ± 4.61	< 0.001*
Residence			< 0.001*
Rural	1,520 (67.02)	1,364 (63.80)	
City/Town	748 (32.98)	774 (36.20)	
Sex			0.6971
Men	1,050 (46.30)	1,078 (50.42)	
Women	1,218 (53.70)	1,060 (49.58)	
Education			< 0.001*
Illiterate	1,108 (48.85)	820 (38,35)	
Literate	1,160 (51.15)	1,318 (61.65)	
Marital status			< 0.001*
Unmarried/Separated/Divorced/Widowed	897 (39.55)	489 (22.87)	
Married	1,371 (60.45)	1,649 (77.13)	
Self-reported quality of life			< 0.001*
Bad	176 (7.76)	282 (13.19)	
Moderate	824 (36.33)	788 (36.86)	
Well	1,268 (55.91)	1,068 (45.95)	
Self-reported health			< 0.001*
Bad	332 (14.64)	620 (29.00)	
Moderate	683 (30.11)	1,080 (50.51)	
Well	1,253 (55.25)	438 (20.49)	
Current smoking			< 0.001*
Yes	502 (22.13)	643 (30.07)	
No	1766 (77.87)	1,495 (69.93)	
Current drinking			< 0.001*
Yes	496 (21.87)	628 (29.37)	
No	1772 (78.13)	1,510 (70.63)	
Physical exercise			< 0.001*
Yes	805 (35.49)	681 (31.85)	
No	1,463 (64.51)	1,457 (68.15)	
Sleeping quality			< 0.001*
Bad	265 (11.68)	329 (15.39)	
Moderate	555 (24.47)	568 (26.57)	
Well	1,448 (63.84)	1,241 (58.04)	
MMSE score			< 0.001*
0–17	144 (6.35)	194 (9.07)	
18–30	2,124 (93.65)	1944 (90.93)	
FI score (mean ± SD)	0.08 ± 0.07	0.10 ± 0.07	< 0.001*

*p < 0.05. Values are *n* (%) unless otherwise noted. MMSE = Mini-Mental State Examination (MMSE), an MMSE score <18 was defined as cognitive impairment. FI: Frailty index.

### 3.2 Frailty trajectory

Three distinct frailty trajectories were identified using the best-fitting trajectory model with a BIC of −19356.99. The APP for every group exceeded 70%, and the proportion of the smallest class comprised over 5% of the total sample. The three trajectories were designated as follows: low-stable group, medium-stable group and low-rapid group. In the low-stable group, participants had a relatively low and stable FI. In the medium-stable group, participants had a moderate FI at baseline with a slight increase over time. And participants in the low-rapid group started with a low FI but experienced a rapid increase over time (9).

### 3.3 Lasso regression

The optimal penalty term lambda was determined using 10-fold cross-validation of the Lasso regression. The model performance was optimal when the lambda.min was set at 0.005471071. Eighteen non-zero coefficients were chosen as potential predictors of frailty progression at this lambda value, including gender, self-reported quality of life, cognitive function, tea consumption, smoking and drinking status, physical labor, social activities, leisure activities, financial support, childhood medical service, age, sleep quality, chewing ability, BMI, and loneliness level. The detailed selection process by Lasso was shown in Supplementary Figures 2, 3.

### 3.4 Multivariate logistic regression and nomogram development

The selected variables were then included in the multivariate logistic regression analysis to further select predictors. Medium-stable trajectory and low-rapid trajectory were distinct patterns of frailty development, so we constructed separate prediction models for these two groups, by using the low-stable group as the reference group. Table 2 displays the odds ratio (OR) (95% confidence interval [CI]) for frailty progression in different trajectories.

**Table 2:** Multinomial logistic regression analysis of FI trajectory groups in older adults.

Variable	Medium-stable group *vs.* low-stable group		Low-rapid group *vs.* Low-stable group	
	OR (95% CI)	*p* value	OR (95% CI)	*p* value
Gender				
Male	Reference		Reference	
Female	5.11 (3.06, 8.55)	<0.001*	1.58 (1.10, 2.25)	0.012*
Self-reported quality of life				
Bad	Reference		Reference	
Moderate	0.70 (0.41, 1.19)	0.184	1.55 (0.82, 2.92)	0.174
Well	0.52 (0.30, 0.91)	0.023*	1.74 (0.91, 3.30)	0.092
MMSE score				
18–30	Reference		Reference	
0–17	6.27 (3.83, 10.28)	<0.001*	1.66 (1.15, 2.41)	0.007*
Drinking tea				
No	Reference		Reference	
Low frequency	0.61 (0.34, 1.11)	0.107	0.79 (0.48, 1.29)	0.342
High frequency	0.68 (0.45, 1.01)	0.056	0.98 (0.72, 1.32)	0.891
Current smoking				
No	Reference		Reference	
Yes	1.84 (1.10, 3.09)	0.021*	0.86 (0.58, 1.29)	0.464
Past drinking				
No	Reference		Reference	
Yes	0.74 (0.46, 1.20)	0.226	0.93 (0.66, 1.31)	0.690
Regular physical labor				
No	Reference		Reference	
Yes	0.38 (0.24, 0.60)	<0.001*	0.91 (0.59, 1.38)	0.644
Social activities				
Low frequency	Reference		Reference	
High frequency	0.19 (0.13, 0.28)	<0.001*	0.58 (0.42, 0.80)	<0.001*
Raise domestic animals/pets				
Low frequency	Reference		Reference	
High frequency	0.64 (0.41, 1.01)	0.053	0.73 (0.53, 1.01)	0.057
Play cards/mah-jongg				
Low frequency	Reference		Reference	
High frequency	0.49 (0.20, 1.18)	0.113	0.98 (0.60, 1.62)	0.946
Watch TV and/or listen to radio				
Low frequency	Reference		Reference	
High frequency	0.64 (0.44, 0.93)	0.019*	0.70 (0.52, 0.95)	0.023*
Financial support				
No	Reference		Reference	
Yes	0.74 (0.49, 1.12)	0.150	0.71 (0.50, 1.01)	0.057
Medical service in childhood				
Inadequate	Reference		Reference	
Adequate	0.62 (0.41, 0.93)	0.020*	0.99 (0.74, 1.34)	0.971
Age	1.70 (0.91, 3.19)	0.099	1.10 (1.08, 1.12)	<0.001*
Sleeping quality				
Bad	Reference		Reference	
Moderate	0.35 (0.21, 0.59)	<0.001*	0.93 (0.55, 1.58)	0.801
Well	0.30 (0.19, 0.49)	<0.001*	1.09 (0.67, 1.76)	0.726
Chewing impairment				
No	Reference		Reference	
Yes	1.69 (0.94, 3.03)	0.078	1.04 (0.75, 1.42)	0.827
BMI				
Normal weight	Reference		Reference	
Underweight	0.80 (0.53, 1.21)	0.281	0.87 (0.62, 1.43)	0.432
Overweight	1.29 (0.75, 2.22)	0.363	0.91 (0.58, 1.43)	0.696
Obesity	2.39 (1.13, 5.08)	0.023*	0.82 (0.38, 1.75)	0.607
Feel lonely				
Low frequency	Reference		Reference	
High frequency	1.77 (1.20, 2.62)	0.004*	1.42 (1.03, 1.97)	0.031*

*p < 0.05. MMSE = Mini-Mental State Examination; MMSE score <18 was defined as cognitive impairment; BMI, Body mass index.

**Figure 1:**
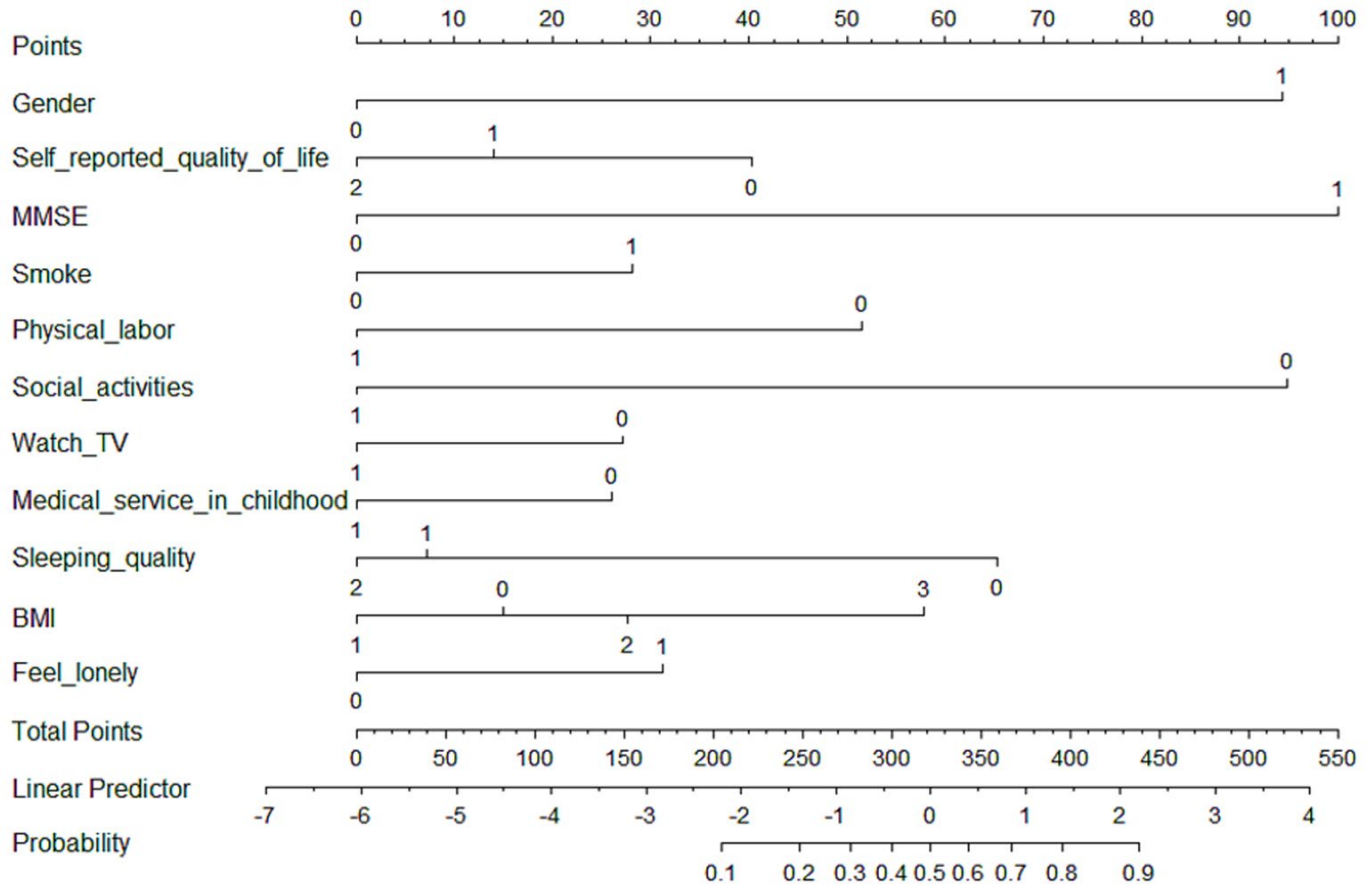
Nomogram for predicting the probability of being in the medium-stable trajectory. The scores corresponding to each predictor were listed at the top of the nomogram. By counting the total score, and projecting it to the bottom risk line, “medium-stable trajectory” probabilities could be estimated.

For medium-stable trajectory, women (OR = 5.11, *p* < 0.001), cognitive impairment (OR = 6.27, *p* < 0.001), smoking (OR = 1.84, *p* = 0,021), obesity (OR = 2.39, *p* = 0.023), and loneliness (OR = 1.77, *p* = 0.004) exacerbated the progression of worsening frailty. While, high quality of life (OR = 0.52), regular physical labor (OR = 0.38) and social activities (OR = 0.19), watching TV (OR = 0.64), adequate childhood medical service (OR = 0.62), and good sleep quality (OR = 0.30) may contribute to decelerating the frailty progression (*p all*<0.001). A nomogram based on these eleven independent features was developed to predict the probability of being in the medium-stable group for old adults (Figure 1). The nomogram illustrated that cognitive function (MMSE) was the strongest predictor of high and progressive risk of frailty, followed by gender and frequency of social participation. The dynamic online nomogram can be accessed at https://online-nomogram.shinyapps.io/Medium_stable_trajectory/. An example was illustrated in Supplementary Figure 4, showing that the individual’s probability of being in the medium-stable trajectory was 96.0%.

**Figure 2:**
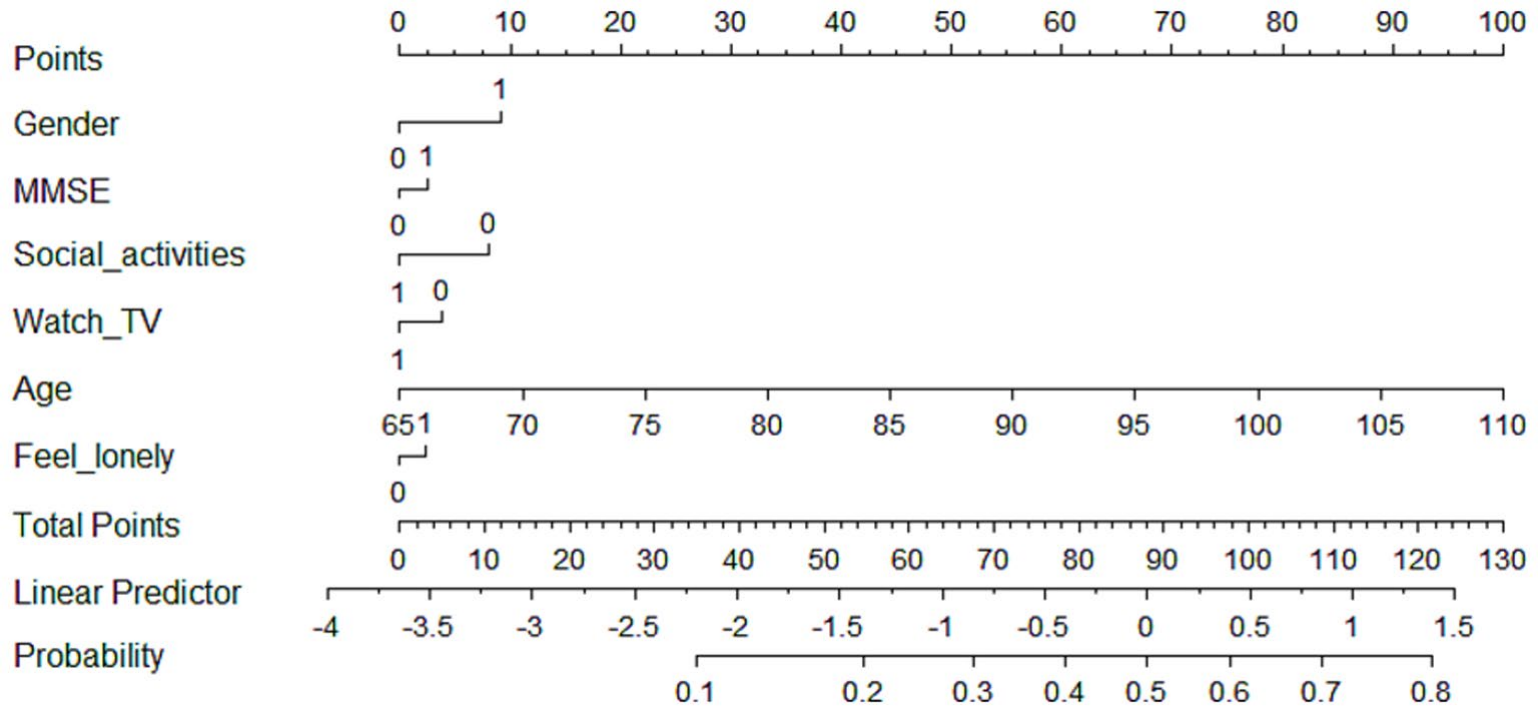
Nomogram for predicting the probability of being in low-rapid trajectory. The scores corresponding to each predictor were listed at the top of the nomogram. By counting the total score, and projecting it to the bottom risk line, “low-rapid trajectory” probabilities could be estimated.

For low-rapid trajectory, six predictive factors including gender, cognitive function, social activities, watching TV, age, and loneliness level were included in the final multivariate model to construct the prediction model, which was visualized as a nomogram (Figure 2). The dynamic nomogram was available online.^1^ Assuming that an 83-year-old woman with cognitive impairment, but regularly participated in social activities and watched TV, and was less likely to feel lonely, the predicted probability was approximately 18.1% (Supplementary Figure 5).

### 3.5 Nomogram validation and evaluation

**Figure 3:**
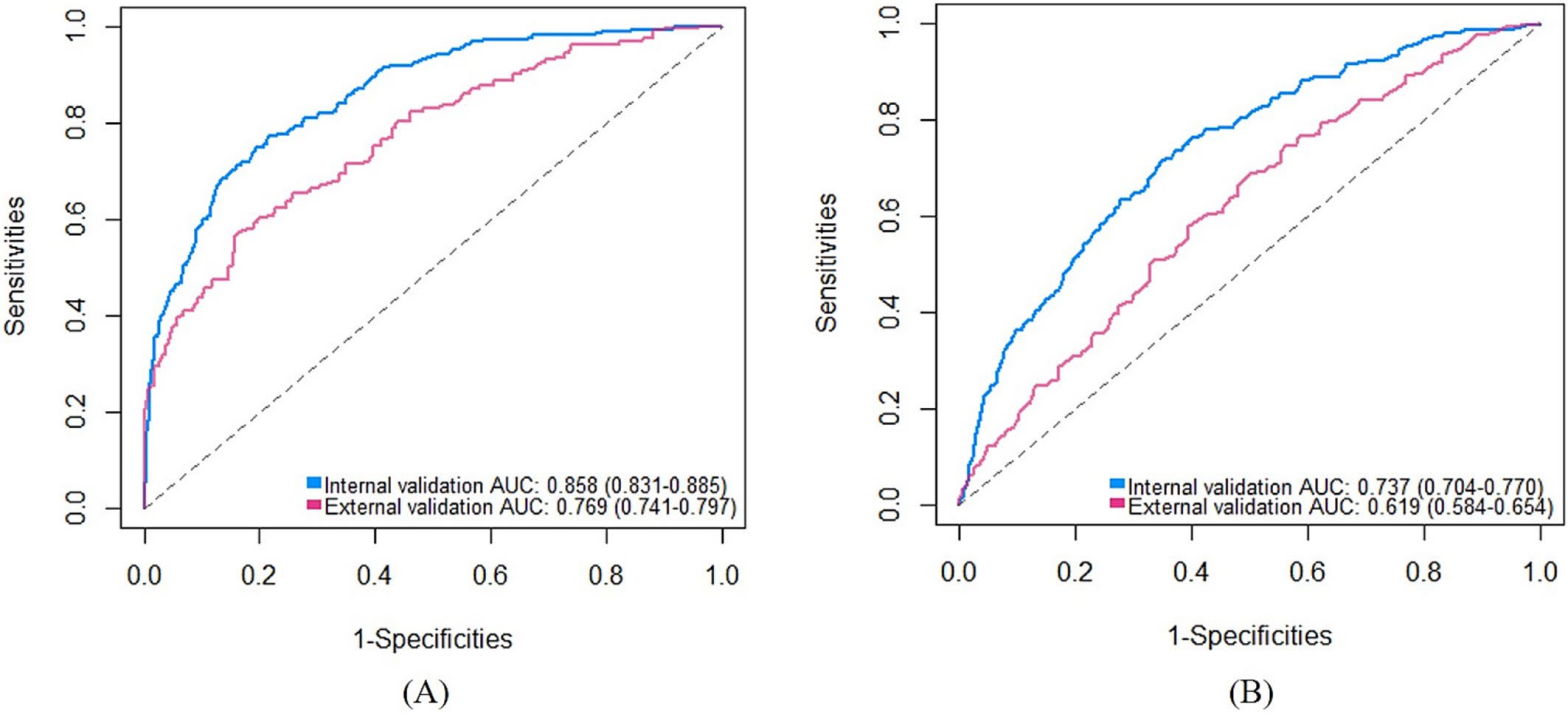
ROC curves of the “medium-stable” nomogram **(A)** and the “low-rapid” nomogram **(B)**. AUC, Area under the receiver operating characteristic curve. ROC, Receiver operating characteristic.

**Figure 4:**
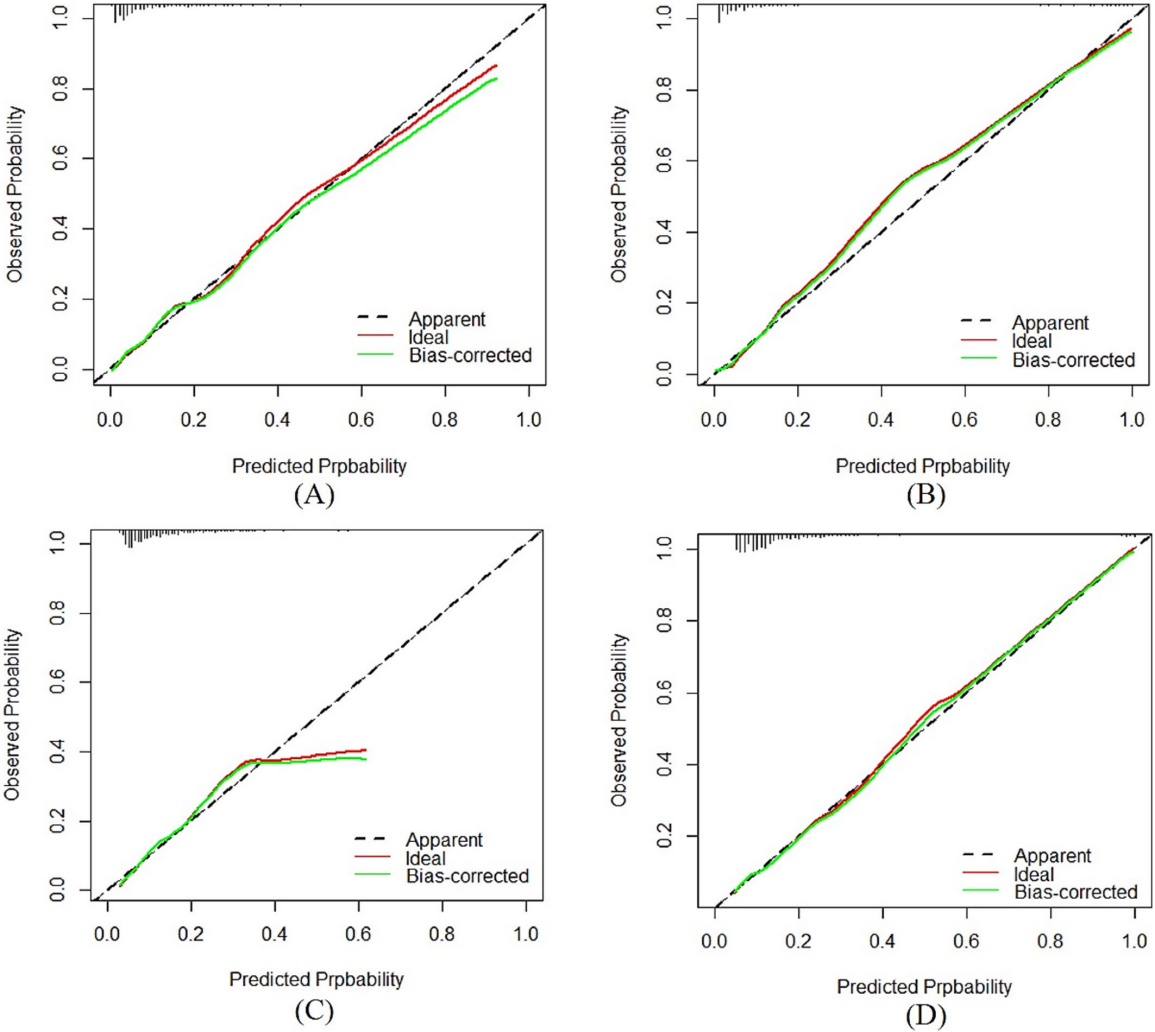
Calibration plots of the “medium-stable” nomogram in the internal **(A)** and external **(B)** validation cohort. Calibration plots of the “low-rapid” nomogram in the internal **(C)** and external **(D)** validation cohort. AUC: The *y* axis indicated the observed incidence for heterogeneous frailty trajectories, and the *x* axis was the predicted probability of heterogeneous frailty trajectories based on the predictive model.

For the medium-stable trajectory, the nomogram showed good discrimination with the C-index of 0.86 in the internal validation cohort and 0.77 in the external validation cohort. The ROC curve of the nomogram also indicated favorable discrimination ability (Figure 3A). And the calibration curves with internal and external validation presented good concordance, as shown in Figures 4A,B. While for the nomogram of the low-rapid trajectory, the C-index was 0.74 for the bootstrapping validation and 0.62 for the external validation cohort. As shown in Figure 3B, ROC analysis presented similar results, with the AUC of the external validation cohort lower than that of the internal validation cohort. However, the calibration curve in the external validation cohort demonstrated that the low-rapid trajectory probabilities predicted by the nomogram agreed well with the actual observation probabilities (Figures 4C,D).

## 4 Discussion

In the current study, nomogram models for predicting heterogeneous frailty progression were developed and validated. Several important factors were identified. Firstly, women, poor quality of sleep and life, cognitive impairment, smoking, no physical and social activities, low childhood medical service, obesity, and loneliness were independent predictors of frailty development in the medium-stable group. Additionally, women, cognitive impairment, lack of social activities, rarely watching TV, advanced age, and loneliness were independent predictors in the low-rapid group. Secondly, based on these findings, the static and dynamic nomograms were constructed for medium-stable group and low-rapid group, respectively. Thirdly, the internal and external validation showed good discrimination and calibration ability of the “medium-stable” nomogram. In contrast, the “low-rapid” nomogram showed acceptable performance in the internal validation cohort but relatively poor discrimination in the external validation cohort.

Our study found that age was a significant risk factor for the deterioration of frailty progression, which is consistent with previous research (23). Frailty is strongly associated with advanced age, primarily as a result of the progressive decline in physiological reserves and the accumulation of age-related pathological changes. These process compromise homeostatic capacity and increase susceptibility to stressors, thereby accelerating frailty development (24). Besides, several physiology and psychosocial factors were also identified as significant predictors. Women were more likely to develop unfavorable frailty trajectories. Previous studies have also found that biological differences (25) and the effects of gender inequality (26) can exacerbate the frailty process for women. Therefore, more attention and health intervention should be devoted to women. Loneliness, sleep, and quality of life are interrelated and directly related to health (27). They all contributed to frailty development in this study, which is consistent with previous research findings (16, 28, 29). Our study also showed that cognitive impairment was associated with an increased risk of frailty progression. It is plausible that cognitive decline and frailty have a common underlying pathology (30). The International Academy on Nutrition and Aging (IANA) and the International Association of Gerontology and Geriatrics (IAGG) defined the simultaneous presence of cognitive impairment and physical frailty as cognitive frailty (31). Regular frailty assessments are recommended for healthcare professionals during disease management of patients with cognitive impairment. On the other hand, cognitively stimulating activities, such as social activities and consumption of intellectually stimulating media, may keep the brain active and reduce the risk of frailty (32, 33).

Furthermore, smoking (34), physical inactivity (35), and obesity (36) were possible risk factors of frailty progression. The rapid progression of frailty with advanced age is associated with increasing physiological dysregulation (37). Specifically, smoking and obesity independently contribute to the dysregulation of inflammatory and metabolic processes, while physical activities improve the function of physiological systems, including muscle, endocrine, and inflammation. These findings underscore the importance of early interventions targeting modifiable lifestyle factors to prevent or slow the deterioration of frailty in aging populations. Lastly, our results demonstrated that the risk of frailty trajectories also involved experiences during early life stages. Consistent with life course theory which posits that childhood conditions can have long-term health consequences, prior studies have reported a positive association between childhood adversity and frailty (38). Notably, other study suggests that older women who received prompt medical care during childhood illness are less likely to experience limitations in daily activities (39). Thus, it is necessary to promote child protection and life course interventions (40).

Besides, a comparative analysis of the two trajectory models reveals both their differences and similarities in risk profiles. Notably, behavior risk factors such as smoking, obesity, and physical inactivity were significant only in the medium-stable nomogram model, suggesting a lifestyle-sensitive path of frailty progression. This finding is consistent with previous studies highlighting the role of modifiable health behaviors in the early stages of frailty (41–43). In contrast, the low-rapid nomogram model did not include these lifestyle variables but still retained cognitive and psychosocial vulnerabilities, possibly reflecting a more biologically driven or less modifiable process (44–46). These differences underscore the heterogeneity in frailty development and suggest that interventions should be tailored accordingly.

Currently, there are relatively few literatures on prediction models of frailty trajectories. More research focused on frailty transition and its risk prediction, with generally short follow-up times (47–51). Using frailty scores at two time points as a criterion for assessing frailty development may fail to capture the longitudinal trajectory and the heterogeneity in frailty progression during follow-up. Besides, there were no internal or external validation of the prediction models of frailty trajectories in some studies (8, 52, 53). Thus, the applicability and generalization of these models in the community population were unknown. For instance, Miao et al. (54) constructed a nomogram for predicting the heterogeneous frailty trajectories among older adults with gastric cancer. However, there was no external validation. In addition, the authors combined two totally different anomalous frailty trajectories for a unified analysis, which would affect the model precision.

Our research has the following advantages. Firstly, frailty trajectories provide more predictive value than frailty states as frailty is a dynamic condition that changes over time. Secondly, we transformed the predictive models into nomograms (including dynamic online nomograms) to quickly calculate the occurrence probability of an individual’s frailty trajectory. Thirdly, external validation was performed using a different set of data with relatively satisfactory results, suggesting that our nomogram models can be generally applied among community-dwelling older adult in China. Fourthly, the identified predictors are easily accessible.

However, some limitations need to be acknowledged. First of all, due to the long observation period of this study, a substantial number of participants who were lost to follow-up or died were excluded, which may have led to an underestimation of frailty levels in Chinese older adults. Besides, most variables and frailty-related indicators were obtained by self-report and there may be information bias. Moreover, predictors cannot explain actual causality. Further empirical studies should be conducted. Last, the discrimination of the “low-rapid” nomogram needs to be improved compared to the “medium-stable” nomogram. And more methods including machine learning methods can be applied to construct the predictive model for better results.

## 5 Conclusion

This study developed and validated two nomogram model for predicting distinct frailty progression trajectories in Chinese older adults. The medium-stable nomogram model and low-rapid nomogram model included eleven and six predictors, respectively. Both models showed acceptable to excellent discriminative performance, with C-index values ranging from 0.62 to 0.86 in internal and external validation. These models can be used for risk assessment to make individualized intervention strategies. Further studies are needed to confirm their efficacy in reducing the risk of frailty progression.

## Data Availability

Publicly available datasets were analyzed in this study. This data can be found at: CLHLS (https://opendata.pku.edu.cn/dataverse/CHADS) and CHARLS (https://charls.charlsdata.com/pages/data/111/zh-cn.html).

## Footnotes

^1^ https://online-nomogram.shinyapps.io/Low_rapid_trajectory/
