# Supplementary material for: Development and validation of nomograms to predict frailty-worsening trajectories among Chinese older adults
Source: Front Public Health. 2025 Jul 17;13:1588303. doi: 10.3389/fpubh.2025.1588303 (PMC12312636; doi:10.3389/fpubh.2025.1588303)
Supplement: Supplementary file 1 [file Table_1.docx]

**Supplementary material**

**Supplementary Table1** Items of the frailty index in CLHLS and CHARLS

| CLHLS | | CHARLS | |
| --- | --- | --- | --- |
| Variables | Deficit values | Variables | Deficit values |
| 1. Bathing | Independently=0; Need some help=0.5; Need help completely=1 | 1. Difficulty with bathing or showering | Yes = 1; No = 0 |
| 2. Dressing | Independently=0; Need some help=0.5; Need help completely=1 | 2. Difficulty with dressing | Yes = 1; No = 0 |
| 3. Toileting | Independently=0; Need some help=0.5; Need help completely=1 | 3. Difficulty with using the toilet | Yes = 1; No = 0 |
| 4. Indoor activities | Independently=0; Need some help=0.5; Need help completely=1 | 4. Difficulty with getting in and out of bed | Yes = 1; No = 0 |
| 5. Eating | Independently=0; Need some help=0.5; Need help completely=1 | 5. Difficulty with eating | Yes = 1; No = 0 |
| 6. Incontinence | Independently=0; Need some help=0.5; Need help completely=1 | 6. Difficulty with controlling urination and defecation | Yes = 1; No = 0 |
| 7. Visit neighbors by oneself | Independently=0; Need some help=0.5; Unable=1 | 7. Difficulty with managing money | Yes = 1; No = 0 |
| 8. Shop by oneself | Independently=0; Need some help=0.5; Unable=1 | 8. Difficulty with shopping for groceries | Yes = 1; No = 0 |
| 9. Cook meals by oneself | Independently=0; Need some help=0.5; Unable=1 | 9. Difficulty with preparing meals | Yes = 1; No = 0 |
| 10. Wash clothing by oneself | Independently=0; Need some help=0.5; Unable=1 | 10. Difficulty with doing housework | Yes = 1; No = 0 |
| 11. Walk continuously for 1 kilometer | Independently=0; Need some help=0.5; Unable=1 | 11. Difficulty with walking 1KM | Yes = 1; No = 0 |
| 12. Lift a weight of 5 kg | Independently=0; Need some help=0.5; Unable=1 | 12. Difficulty with lifting or carrying weights over 5 kg | Yes = 1; No = 0 |
| 13. Continuously crouch and stand up 3 times | Independently=0; Need some help=0.5; Unable=1 | 13. Difficulty with stooping, kneeling, or crouching | Yes = 1; No = 0 |
| 14. Use public transportation by oneself | Independently=0; Need some help=0.5; Unable=1 | 14. Difficulty with using phone calls | Yes = 1; No = 0 |
| 15. Put hand behind neck | Both hands=0; Only one hand=0.5; Neither=1 | 15. Difficulty with reaching arms above shoulder level | Yes = 1; No = 0 |
| 16. Put hand behind lower back | Both hands=0; Only one hand=0.5; Neither=1 |  | |
| 17. Raise arm upright | Both hands=0; Only one hand=0.5; Neither=1 |  |  |
| 18. Stand up from sitting in a chair | Yes=0, without using hands; Yes, using hands=0.5; Cannot=1 | 16. Difficulty with getting up from a chair after sitting for long periods | Yes = 1; No = 0 |
| 19. Pick up a book from the floor | Yes, standing=0; Yes, sitting=0.5; Cannot=1 | 17. Difficulty with picking up a coin from the table | Yes = 1; No = 0 |
| 20. Hearing impairment | No=0; Yes=1 | 18. Self-reported hearing problems | Yes = 1; No = 0 |
| 21. Visual impairment | No=0; Yes=1 | 19. Self-reported vision problems | Yes = 1; No = 0 |
| 22. Self-reported health | Very good/Good=0; Moderate=0.5; Bad=0.75; Very bad=1 | 20. Self-reported general health status | Poor =1;  Fair = 0.75;  Good = 0.5;  Very good=0.25;  Excellent=0 |
| 23. Health status compared with last year | Much /Slightly better=0; Almost the same=0.5; Slightly worse=0.75; Much worse=1 | 21. Depression: CESD-10 questionnaire | CESD-10 >10 =1; CESD-10≤10 =0 |
| 24. Interviewer-rated health | Surprisingly healthy=0; Relatively healthy=0.5; Moderately ill=0.75; Very ill=1 |  | |
| 25. of times suffering from serious illness within the past two years | 0 time=0; 0ne time=1; Two or more times=2 |  |  |
| 26. Suffering from hypertension | No=0; Yes=1 | 22. Self-reported physician diagnosed hypertension | Yes = 1; No = 0 |
| 27. Suffering from diabetes | No=0; Yes=1 | 23. Self-reported physician diagnosed diabetes | Yes = 1; No = 0 |
| 28. Suffering from heart disease | No=0; Yes=1 | 24. Self-reported physician diagnosed heart disease | Yes = 1; No = 0 |
| 29. Suffering from stroke, CVD | No=0; Yes=1 | 25. Self-reported physician diagnosed stroke | Yes = 1; No = 0 |
| 30. Suffering from bronchitis, emphysema, pneumonia, asthma | No=0; Yes=1 | 26. Self-reported physician diagnosed asthma | Yes = 1; No = 0 |
| 31. Suffering from tuberculosis | No=0; Yes=1 | 27. Self-reported physician diagnosed chronic lung disease | Yes = 1; No = 0 |
| 32. Suffering from cancer | No=0; Yes=1 | 28. Self-reported physician diagnosed cancer | Yes = 1; No = 0 |
| 33. Suffering from gastric or duodenal ulcer | No=0; Yes=1 | 29. Self-reported stomach or other digestive disease (except for tumor or cancer). | Yes = 1; No = 0 |
| 34. Suffering from Parkinson’s disease | No=0; Yes=1 | 30. Self-reported physician diagnosed any emotional, nervous, or psychiatric problems | Yes = 1; No = 0 |
| 35. Suffering from dementia | No=0; Yes=1 | 31. Self-reported physician diagnosed memory-related disease | Yes = 1; No = 0 |
| 36. Suffering from arthritis | No=0; Yes=1 | 32. Self-reported physician diagnosed arthritis | Yes = 1; No = 0 |
| 37. Suffering from bedsore | No=0; Yes=1 | 33. Self-reported liver disease | Yes = 1; No = 0 |
| 38. Suffering from cataracts | No=0; Yes=1 | 34. Self-reported dyslipidemia | Yes = 1; No = 0 |

*CLHLS: Chinese Longitudinal Healthy Longevity Survey. CHARLS: China Health and Retirement Longitudinal Study.

*Depression was evaluated using Center for Epidemiologic Studies Depression Scale (CESD). In the CHARLS, CESD-10 was used. The total score ranges from 0 to 30. The higher score indicates more severe depressive symptoms.

*We used the sum of deficit points present in the individual to divide the total number of deficits considered as an indicator of frailty index (FI). If there were missing items (allowed within 1/4 of total items), then will be excluded from both the denominator and the numerator. It is generally accepted that ≥0.25 indicates frailty.

**Supplementary Table 2** Measurement of variables in CLHLS 2008

| **Variables** | **Measurement** |
| --- | --- |
| **Sociodemographic Characteristics** |  |
| Age | Continuous variable |
| Gender | 0=Male, 1=Female |
| Education | 0= Illiterate, 1= Literate |
| Marital status | 0=Unmarried/Separated/Divorced/Widowe, 1=Married |
| Residence | 0=Rural, 1=City/Town |
| Co-residence | 0=Living alone, 1=Living with household members |
| Primary occupation | 0=labor work,1=Mental work |
| Economic status | 0=Low level, 1=Average level,  2=High level |
| Financial support can pay for daily expenses | 0=No, 1=Yes |
| Severe disease insurance | 0=No, 1=Yes |
| Public old-age insurance | 0=No, 1=Yes |
| Medical service in childhood | 0=Inadequate, 1=Adequate |
| Childhood starvation | 0=No, 1=Yes |
| Self-reported quality of life | 0=Bad, 1=Moderate, 2=Well |
| Lose mother before aged 18 | 0=No, 1=Yes |
| Lose father before aged 18 | 0=No, 1=Yes |
| Father's main occupation in childhood | 0=labor work,1=Mental work |
| **Lifestyles** |  |
| Staple food | 1=Rice, 2= Corn，3= Wheat, 4= Rice and wheat, 5=Other |
| Regular fruit intake | 0=Low frequency,  1=High frequency |
| Regular vegetables intake | 0=Low frequency,  1=High frequency |
| Current smoking | 0=No, 1=Yes |
| Past smoking | 0=No, 1=Yes |
| Current drinking | 0=No, 1=Yes |
| Past drinking | 0=No, 1=Yes |
| Current drinking tea | 0=No, 1= Low frequency,  2=High frequency |
| Current exerciser | 0=No, 1=Yes |
| Ex-exerciser | 0=No, 1=Yes |
| Regular physical labor | 0=No, 1=Yes |
| Housework | 0= Low frequency, 1= High frequency |
| Do field work | 0= Low frequency, 1= High frequency |
| Garden work | 0= Low frequency, 1= High frequency |
| Read newspapers/books | 0= Low frequency, 1= High frequency |
| Raise domestic animals/pets | 0= Low frequency, 1= High frequency |
| Play cards/mah-jongg | 0= Low frequency, 1= High frequency |
| Watch TV or listen to radio | 0= Low frequency, 1= High frequency |
| Social activities | 0= Low frequency, 1= High frequency |
| Religious activities | 0= Low frequency, 1= High frequency |
| Sleeping quality | 0=Bad, 1=Moderate, 2=Well |
| **Physical and mental condition** |  |
| Feel lonely | 0= Low frequency, 1= High frequency |
| Cognitive impairment | 0=No, 1=Yes |
| Chewing impairment | 0=No, 1=Yes |
| BMI* | 0= Underweight, 1= Normal weight,  2=Overweight, 3= obese |
| **Community service** |  |
| Personal care services | 0=Unavailable, 1= Available |
| Home visit services | 0=Unavailable, 1= Available |
| Psychological services | 0=Unavailable, 1= Available |
| Daily shopping services | 0=Unavailable, 1= Available |
| Social and recreation services | 0=Unavailable, 1= Available |
| Legal aid services | 0=Unavailable, 1= Available |
| Healthcare education services | 0=Unavailable, 1= Available |
| Neighborhood-relation services | 0=Unavailable, 1= Available |

*CLHLS: Chinese Longitudinal Healthy Longevity Survey. BMI: Body mass index.

*Cognitive function was measured by the Chinese version of Mini-Mental State Examination (MMSE)，an MMSE score of <18 was defined as cognitive impairment.


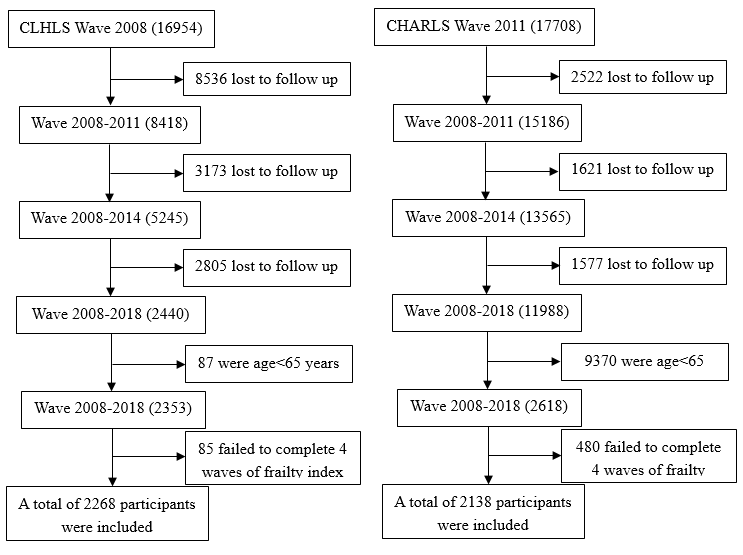


**Supplementary Figure 1.** Flow chart of sample selection from CLHLS and CHARLS.


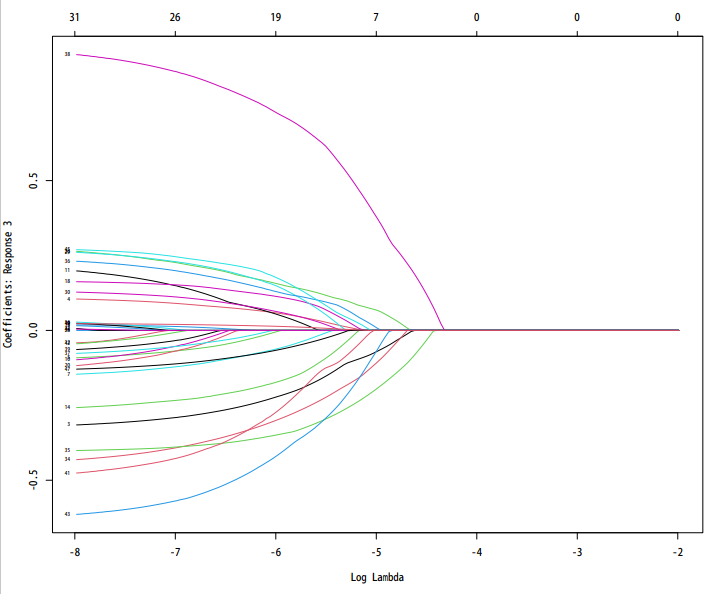


**Supplementary Figure 2.** Lasso coefficient path diagram.

Note: Each curve represented the trajectory of each coefficient of the independent variable.


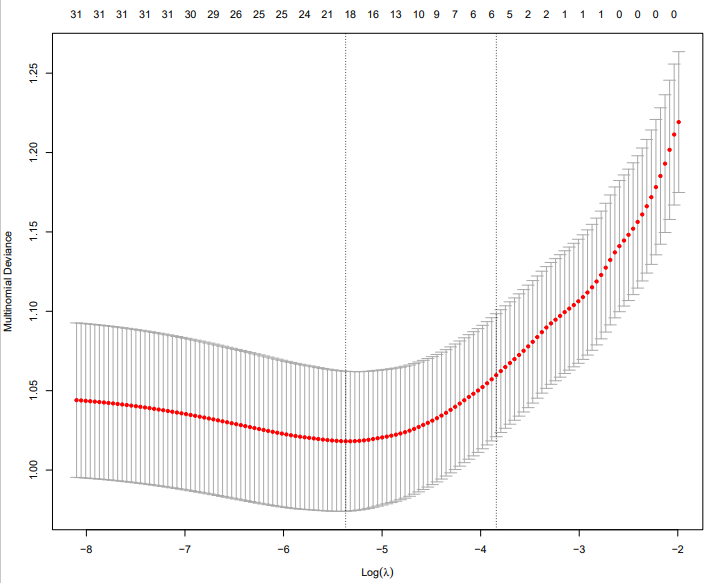


**Supplementary Figure 3.** Lasso Cross-Validation Curve.

Note: Identification of the optimal penalization coefficient (λ) in the LASSO model was achieved by 10-fold cross-validation and the minimum criterion. The dashed line on the left represents the log (λ) value corresponding to the minimum deviance, i.e., lambda.min. The dashed line on the right represents the log (λ) value corresponding to one standard error away from the minimum deviance, i.e., lambda.lse.


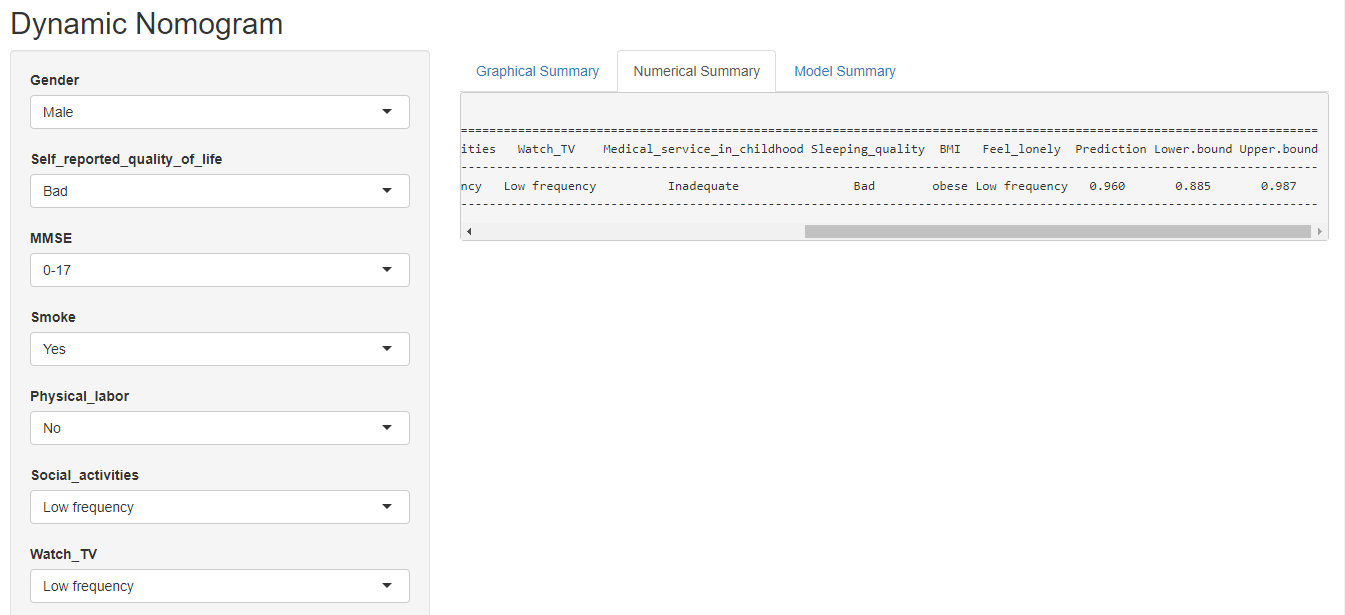


**Supplementary Figure 4.** The web-based dynamic online nomogram in medium-stable trajectory (<https://online-nomogram.shinyapps.io/Medium_stable_trajectory/>). Note: This figure depicted an example for predicting the probability of being in the medium-stable trajectory according to the following eleven predictors: gender (male), self-reported quality of life (bad), MMSE (0-17), smoke (yes), physical labor (no), social activities (low frequency), watch TV (low frequency), medical service in childhood (inadequate), sleeping quality (bad), BMI (obese), feel lonely (low frequency), the predicted probability was 96.0%.


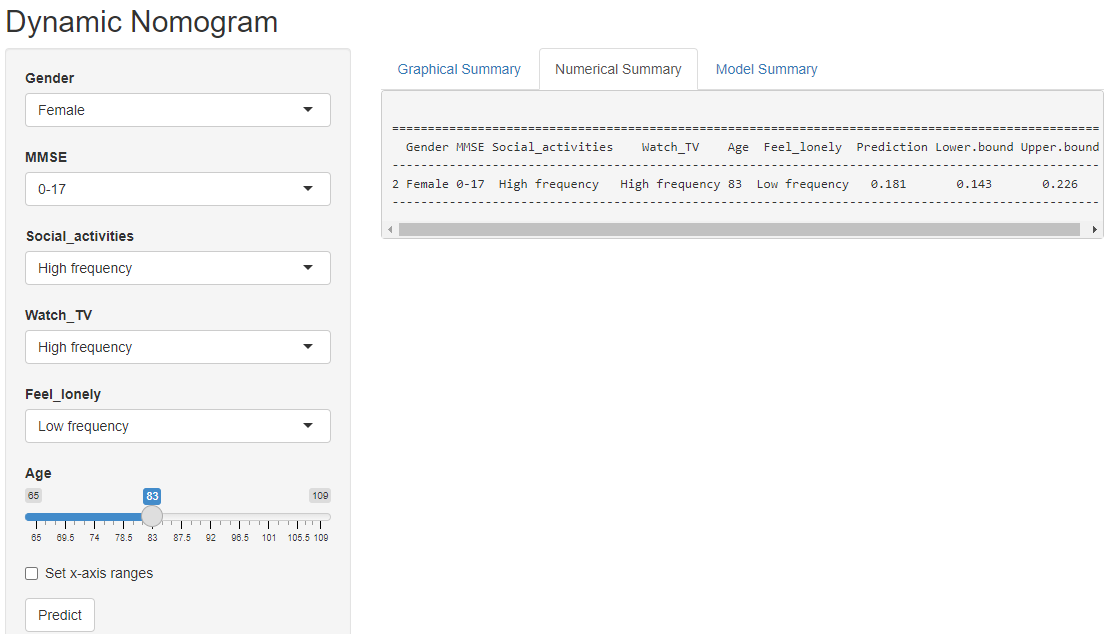


**Supplementary Figure 5.** The web-based dynamic online nomogram in low-rapid trajectory (<https://online-nomogram.shinyapps.io/Low_rapid_trajectory/>).

Note: This figure depicted an example for predicting the probability of being in the low-rapid trajectory according to the following six predictors: gender (female), MMSE (0-17), social activities (high frequency), watch TV (high frequency), feel lonely (low frequency), age (83 years old), the predicted probability was 18.1%.
